# Toxicological studies on the botanical supplement LI12542F6 containing extracts of *Sphaeranthus indicus* flower heads and *Mangifera indica* (mango tree) bark

**DOI:** 10.1002/fsn3.931

**Published:** 2019-01-29

**Authors:** Earle R. Nestmann, Venkata Krishnaraju Alluri, Sundararaju Dodda, Barbara A. Davis

**Affiliations:** ^1^ Health Science Consultants Mississauga Ontario Canada; ^2^ Laila Nutraceuticals R&D Center Vijayawada India; ^3^ PLT Health Solutions Inc. Morristown New Jersey

**Keywords:** Ames bacterial reverse mutation assay, LI12542F6, mammalian erythrocyte micronucleus test, *Mangifera indica*, Myotor, repeat dose 90‐day subchronic toxicity study, *Sphaeranthus indicus*

## Abstract

LI12542F6, a botanical extract composed of *Sphaeranthus indicus* and *Mangifera indica*, was evaluated for mutagenicity in bacteria*,* clastogenicity in mouse bone marrow, acute oral and dermal toxicity in the rat, irritation (dermal, eye) in rabbit, and subacute and subchronic toxicity (28 and 90 days) in the rat. All studies followed standard OECD test protocols, in accordance with the principles of Good Laboratory Practice (GLP). LI12542F6 did not induce mutations in the bacterial assay using *Salmonella* and *Escherichia coli* strains, nor did it induce genotoxic effects in erythrocytes from mouse bone marrow. LI12542F6 was found to have oral and dermal LD_50_ values greater than the limit dose of 2,000 mg/kg body weight in the rat. In an eye irritation/corrosion test, LI12542F6 caused conjunctival redness, corneal opacity, and chemosis and is classified as Category 2A (“irritating to eyes – reversible eye effect”). Doses in the 28‐day and 90‐day rat oral toxicity studies were 0, 500, 1,000, and 1,500 and 0, 1,000, 1,500, and 2,000 mg/kg body weight/day, respectively, administered by gavage. Both studies featured a recovery period. Minor effects were random and not treatment related except for local irritation of the forestomach in the 28‐day study, evidenced by histopathologic examination, in mid‐ and high‐dose animals. The frequency and severity of these effects were reduced in the recovery group; irritation was not found in the forestomach of rats in the 90‐day study. The no observed adverse effect level (NOAEL) was greater than the highest dose tested, that is, >2,000 mg/kg in the 90‐day study. This botanical composition will be marketed commercially for muscle health as Myotor™.

**Abbreviations**
ANOVA: analysis of variance
CMC‐Na: carboxymethylcellulose sodium
FOB: functional observational battery
GHS: globally harmonized system
GLP: Good Laboratory Practice
LD_50_: lethal dose 50 percent
NOAEL: no observed adverse effect level
OECD: Organization for Economic Co‐operation and Development
PCE: polychromatic erythrocytes
UHPLC: ultra‐high performance liquid chromatography

## 1 INTRODUCTION

LI12542F6 is a proprietary blend of two botanicals, *Sphaeranthus indicus* Linn. (family Asteraceae) flower head extract and *Mangifera indica* Linn. (family Anacardiaceae) (mango tree) bark extract. Both have histories of use in ancient Indian Ayurvedic medicine for various conditions (Ramachandran, 2013; Sharma, Yeine, & Dennis, 2001). *Mangifera indica* (mango tree) is a native plant of the Indian subcontinent that is now naturalized in many tropical regions across the globe (Batool et al., 2018). Mango tree bark preparations have been used widely as folk medicines in tropical and subtropical regions (Coe & Anderson, 1996). The glucosyl xanthone, mangiferin, found in mango fruit, leaves, and bark among other plants, has been reported to have antioxidant, antidiabetic, immunomodulatory, antigenotoxic, and anti‐inflammatory properties (Prado, Merino, & Acosta, 2015). The bark and leaves of *M. indica* are rich in mangiferin (Yoshimi et al., 2001), with the bark containing approximately 20% mangiferin, whereas the leaf extract contains around 7% mangiferin (unpublished observations from the laboratory of VKA). As such, an aqueous mango bark extract with the trade name Vimang^®^ is used as a nutraceutical in Cuba for patients with elevated stress and other disorders (Guevara, Riaño, & Alvarez, 2004).

*Sphaeranthus indicus* is an aromatic herb, distributed widely in plains throughout India (Ramachandran, 2013). Individual parts or the plant in its entirety are used for managing a variety of ailments owing to a multitude of reported functions. Most notable are immunomodulatory, hepatoprotective, analgesic, antidiabetic, antioxidant, anxiolytic, anti‐inflammatory, and antihyperlipidemic activities. *Sphaeranthus indicus* is a component of a dietary supplement preparation that in combination with *Garcinia mangostana* has demonstrated efficacy in weight and blood lipid management (Stern, Peerson, Mishra, Mathukumalli, & Konda, 2013).

There are a number of toxicological studies on extracts and preparations of *M. indica,* including the active ingredient mangiferin (Garrido, Rodeiro, & Hernández, 2009; Prado et al., 2015; Rodeiro, Cancino, & González, 2006; Rodeiro, Hernandez, & Morffi, 2012), and of *S. indicus* (Ambikar & Mohanta, 2013; Nahata & Dixit, 2011; Saiyed, Sengupta, & Krishnaraju, 2015). It is important also to understand, using experimental toxicology studies, whether there are toxicological concerns related to the combination. For that reason, we evaluated the *M. indica* and *S. indicus* extract preparation, referred to as LI12542F6, in a complement of in vitro and in vivo toxicology models.

The purpose of the present paper was to publish new toxicology data to establish a robust database for the use of LI12542F6 in health products intended for human consumption. GLP (Good Laboratory Practice) study reports are described below. Toxicological evaluation of the botanical combination LI12542F6, which will be available commercially as Myotor™, is important to confirm safety.

## 2 MATERIALS AND METHODS

### 2.1 Laboratories

GLP tests were carried out in the test facilities of Advinus Therapeutics Limited in Bengaluru, India, and of Laila Nutraceuticals R & D in Vijayawada, Andhra Pradesh, India, on behalf of study sponsor Laila Nutraceuticals, also of Vijayawada, Andhra Pradesh, India.

Both facilities are GLP‐compliant laboratories in accordance with the OECD Principles of Good Laboratory Practice (GLP) (OECD Principles of Good Laboratory Practice, 2008), certified by the National GLP Compliance Monitoring Authority (NGCMA) of India. All studies described in this paper were audited and verified by the respective laboratory's Quality Assurance Unit.

### 2.2 Test material

The test material LI12542F6 is a novel herbal formulation for enhancing muscle growth, endurance, and physical performance. It is a composition, containing 65% (w/w) of a blend of the extracts of *Sphaeranthus indicus* L. (family Asteraceae) flower heads and *Mangifera indica* L. (family Anacardiaceae) stem bark at a 2:1 ratio and 35% (w/w) of excipients. Both raw materials are widely distributed throughout the Indian subcontinent. *Sphaeranthus indicus* flower head raw material was collected from wild crafted source from the state of Orissa, India, and *Mangifera indica* bark was procured from plantation source in Andhra Pradesh, India. The raw materials were identified by a qualified taxonomist and compared with the authentic raw materials (RDM) and the voucher specimens (#s 6578 for *S. indicus* and 6246 for *M. indica*), preserved in the Taxonomy Division of Laila Nutraceuticals R & D Center, Vijayawada, India.

The dried raw materials of *Sphaeranthus indicus* and *Mangifera indica* were pulverized and processed individually using methanol/ethyl acetate and aqueous methanol, respectively, as solvent media, to obtain the corresponding extracts. These extracts were concentrated separately under vacuum and blended along with the excipients. The mixture was pulverized, sieved, and blended to obtain LI12542F6 as a free‐flowing powder. The blend was standardized to contain not less than 4% of 7‐hydroxyfrullanolide and 2.5% of mangiferin, which are the phytochemical reference markers of *S. indicus* and *M. indica*, respectively. The blend was assigned a shelf life of 2 years, based on the data from accelerated and real‐time stability studies. The test material for all the studies described in the paper was provided by the sponsor Laila Nutraceuticals, Vijayawada, India, along with an authorized “Certificate of Analysis.” Each lot of LI12542F6 was thoroughly analyzed for compliance with the predefined specifications for the blend. For repeated dose toxicity studies, the test material dose formulation was evaluated for concentration, stability, and homogeneity using a validated analytical method prior to dosing of the experimental animals. LI12542F6 is available from PLT Health Solutions (Morristown, NJ) as Myotor™.

### 2.3 Analytical procedure

Analysis of LI12542F6 was carried out using ultra‐high performance liquid chromatography (UHPLC; Waters, Acquity) system equipped with a thermostat‐controlled column oven compartment, autosampler, photodiode array detector, and Empower 2 software (Waters Corporation, Milford, MA). The sample preparation involves extraction of the sample using aqueous methanol, followed by filtration through 0.22‐μm PVDF filter. The sample solution was analyzed using Waters X Bridge C18 column 3.5 μm (100 × 4.6 mm).

**Figure 1:**
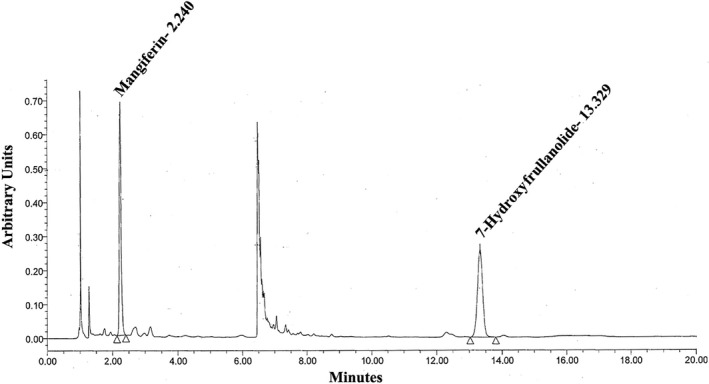
A representative high performance liquid chromatography depicts the phytochemical markers of LI12542F6. The peaks eluted at 2.24 and 13.329 min are identified as mangiferin and 7‐hydroxyfrullanolide, respectively. The elution profile is plotted in arbitrary units versus elution time (min)

A gradient elution system consisting of solvent A (0.1% v/v orthophosphoric acid in water) and solvent B (acetonitrile) as mobile phase at 1.0 ml/min was used with the run starting at sample injection, with a mixture of 87% A and 13% B as initial eluent for 5 min, then increased the gradient to 65% A and 35% B in 0.1 min, finally maintained an isocratic run at 65% A, 35% B for further 14 min. A typical HPLC of LI12542F6 is depicted in Figure 1. The representative chromatogram shows mangiferin and 7‐hydroxyfrullanolide as two peaks eluted at 2.24 and 13.33 min, respectively, at 210 nm. Identification of these peaks was carried out using the respective pure phytochemical reference standards.

### 2.4 Chemicals, reagents, and bacterial strains

Bacto agar was procured from Difco Laboratories Ltd, Becton Dickinson (Sparks, USA). Clinical chemistry reagents and the reagents required for hematology analyses were purchased from Instrumentation Laboratories (Bedford, MA) and Siemens Healthcare GmbH (Erlangen, Germany), respectively. Carboxymethylcellulose, sodium (CMC‐Na), polyethylene glycol 400 (PEG 400), nicotinamide adenine dinucleotide phosphate (NADP) and glucose‐6‐phosphate (G6P), sodium azide, 2‐nitrofluorene, 9‐aminoacridine, 4‐nitroquinoline‐N‐oxide and 2‐aminoanthracene, fetal bovine serum (FBS), cyclophosphamide monohydrate (CPA) were obtained from Sigma Chemicals (St Louis, MO, USA). *Salmonella typhimurium* strains TA 98, TA100, TA 1535, and TA 1537 were obtained from NCTC (Salisbury, UK). *Escherichia coli*, WP2 *uvrA* (pKM101), was obtained from NCIMB (Aberdeen, Scotland). Their identity and characteristics were verified prior to testing. Lyophilized rat liver S9 fraction induced by Aroclor 1254 was purchased from Celsis In vitro Technologies (Baltimore, MD). Absolute methanol, dimethylsulfoxide (DMSO), sodium thiopentone barbiturate, isoflurane, magnesium chloride (MgCl_2_), potassium chloride (KCl), and sodium phosphate salts were purchased from Sisco Research Laboratories Pvt. Ltd (Mumbai, India).

#### 2.4.1 In vitro genotoxicity

##### Bacterial reverse mutation test

The *Salmonella* mammalian microsome reverse mutation test protocol conformed to the appropriate OECD Guideline 471 (1997), which is based on the “Ames test” as described by Maron and Ames (1983) and was compliant with GLP.

LI12542F6 was found to be uniformly dissolved in DMSO. The doses of 0, 50, 100, 200, 400, 800, 1,600, 3,200, and 5,000 μg/plate were selected to evaluate toxicity in strain TA100 with the presence and absence of S9. Slight cytotoxicity was found at 5,000 μg/plate (‐S9), and the maximum dose was 5,000 for both the initial plate incorporation test and the confirmatory preincubation assay.

Bacterial strains were grown overnight in Oxoid Nutrient Broth No. 2. For the toxicity and initial mutation tests, the bacteria were combined with test material (±S9) in soft overlay agar containing biotin–histidine/tryptophan, and then plated on hard agar. Test doses in the initial plate incorporation assay were 0, 50, 158, 500, 1,581, and 5,000 μg/plate. For the confirmatory, preincubation assay, the bacterial cells were combined with test material, ±S9, incubated with shaking for 20 min and then soft agar was added before plating over hard agar. Test doses were 0, 100, 266, 707, 1,880, and 5,000 μg/plate. Plates in triplicate were incubated at 37°C for approximately 67 hr, the revertant colonies were counted, and background growth was examined as an indicator of cytotoxicity.

#### 2.4.2 In vivo genotoxicity

##### In vivo mammalian micronucleus test

The test protocol conforms to the appropriate OECD Guideline 474 (2016) and was performed according to GLP using male and female Swiss albino mice [(five groups of 10 mice (5M + 5F)]. The groups included a negative control, three treatment groups (oral doses of 500, 1,000, or 2,000 mg/kg body weight), and a positive control (cyclophosphamide monohydrate at 40 mg/kg body weight by intraperitoneal injection). The vehicle for the test material was 0.5% (w/v) CMC‐Na. Doses of test article were administered by gavage (10 ml/kg bw) to the treatment groups on day 1 and again 24 hr later. The positive control group was given only one dose of cyclophosphamide monohydrate on the second day. On the third day (at 22–24 hr after second‐day treatment), bone marrow was removed from the femurs of the euthanized animals and centrifuged to concentrate the blood cells. Cells were smeared onto microscope slides (three per animal), fixed and stained with 6% Giemsa.

To determine cytotoxic potential, the proportion of immature or polychromatic erythrocytes among the total of 500 polychromatic and normochromatic erythrocytes was determined per animal. Microscopic examination of at least 4,000 immature erythrocytes determined the percent incidence of micronucleated polychromatic erythrocytes (PCEs).

#### 2.4.3 In vivo toxicity studies

##### Acute oral toxicity

LI12542F6 was tested, in accordance with GLP, in the female rat to determine its potential to cause acute lethality, using the “up‐and‐down procedure” described in Guideline 425 of the OECD (2008). As LI12542F6 is not expected to have potential toxicity, the intent of the present study was to test at the maximum dose of 2,000 mg/kg body weight. The vehicle for the test material was 0.5% (w/v) CMC‐Na. Doses were administered by gavage (10 ml/kg bw). Following dosing, the animals were observed for morbidity and mortality for 14 days.

##### Acute dermal toxicity

Using the limit test procedure, OECD Guideline 402 (1987), 10 animals (5M and 5F) were treated with 2,000 mg/kg body weight. Test animals were Wistar rats procured from Vivo Bio Tech Ltd. (Telangana, India). LI12542F6 (2000 mg/kg body weight) was moistened with 3 ml of distilled water for application to an area of 5 × 6 cm of shaved skin and held in place for 24 hr by a porous gauze dressing before being cleaned from the skin to remove residual material. Following dermal administration, observations were made after 30 min, 1, 2, 3, and 4 hr on day 0 and daily afterward for 14 days. Followed by sacrifice, all the animals were subjected to gross pathological examination.

##### Acute dermal irritation/corrosion

For evaluation of dermal irritation, test material LI12542F6 (0.5 g) was moistened with 3 ml of distilled water and applied to clipped, intact skin (6 cm^2^ of left flank) of rabbit test animals. The negative control of 3‐ml distilled water was applied to clipped intact skin (6 cm^2^ of right flank).

On the day of treatment, gauze patches were applied for a 4‐hr exposure period after which residual test material was rinsed away with distilled water without altering the existing response. In the initial test, one New Zealand white rabbit (Mahaveera Enterprise, Hyderabad, Telangana, India) was treated with the test material (OECD Guideline 404, 2015). As no severe irritation/corrosion of the skin was noted after 72 hr, a confirmatory test proceeded with two additional animals at the same time. Observations were made 1, 24, 48, and 72 hr and days 7 and 14 (initial test) or days 7 and 10 (confirmatory test), and irritation was scored according to GHS (2015).

##### Acute eye irritation/corrosion

Treatment consisted of instillation of 100 mg of ground test material into the left conjunctival sac which was slightly pulled away from the eyeball to form a space and to allow easy delivery of test material into the sac as per OECD Guideline 405 (2012). The eyelids were held together for a few seconds to prevent the removal of test material. After 1‐hr treatment, the eye was rinsed with saline. The right eye, which remained untreated, served as control.

A total of three rabbits per test group were subjected to a rigorous study of ocular structures, the cornea, iris, and conjunctiva. In the initial test, a single New Zealand white rabbit (Mahaveera Enterprise, Hyderabad, Telangana, India) was treated. After grading the treated eye for severity of effects at 1, 24, 48, and 72 hr posttreatment and finding no severe effects, the confirmatory test proceeded with 2 additional rabbits. Scoring of eye reactions was determined in a dark room with the use of an ophthalmoscope at 1, 24, 48, and 72 hr, and day 7 for the initial test; at 1, 24, 48, and 72 hr and days 7, 14, and 21 for the confirmatory test to assess the reversibility of the effects. Scores for corneal opacity, iris, conjunctivae, and chemosis were averaged for three scoring times (24, 48, and 72 hr) according to harmonized classification guidelines (GHS, 2015). Fluorescent ophthalmic strips were used to evaluate eye lesions. Finally, animals were weighed at the end of the study to compare the variations in this parameter.

##### 28‐day repeated dose toxicity study

As per OECD Guideline 407 (2008), ten Wistar rats (5M + 5F) (Vivo Bio Tech Ltd, Telangana, India) were used in each of six groups, including a vehicle control (GC); low, mid, and high treatment groups (GL 500 mg/kg; GM 1,000 mg/kg; and GH 1,500 mg/kg, respectively); and two recovery groups (GCR control and GHR high dose). The vehicle for the test material was 0.5% (w/v) CMC‐Na. Doses were administered by gavage (10 ml/kg bw). Dose verification and homogeneity were analyzed using HPLC by the laboratory on day 1 and day 25 and found to be within the acceptable limits.

All animals were treated for 28 days and the recovery groups continued on test without treatment for an additional 14 days. Animals were observed twice daily for morbidity and mortality, and cage side evaluations of general clinical signs were made daily. Examination of detailed clinical signs was made pretreatment and after each week. Ophthalmic examination took place prior to treatment for all the groups and, during week 4 of the treatment period and week 6 for the recovery groups. Body weight and feed consumption were determined weekly. Clinical pathology examinations took place on day 29 for main groups and day 43 for the recovery groups. Blood was collected after overnight fasting (water ad libitum) for hematology and clinical chemistry analysis. Hematological parameters included red and white blood cells; neutrophils; lymphocytes; monocytes; eosinophils; basophils; large unstained cells; reticulocytes; hemoglobin; hematocrit; mean corpuscular (volume. hemoglobin and hemoglobin concentration); platelets; and clotting time. Clinical chemistry parameters were glucose; blood urea nitrogen; urea; creatinine; total cholesterol; triglycerides; total bilirubin; aspartate aminotransferase; alanine aminotransferase; alkaline phosphatase; total protein; albumin; calcium; phosphorus; sodium; potassium; and chloride. Following sacrifice, all animals were subjected to external and internal gross pathological examination. Subsequently, various organs and tissues were collected for weighing (adrenal glands, brain, epididymides, heart, kidneys, liver, ovaries, prostate gland with seminal vesicles and coagulating glands, spleen, testes, thymus, and uterus with cervix). Tissues and organs for histopathological investigation included these plus aorta, cecum, colon, duodenum, esophagus, eyes, gross lesions, femur (bone with joint), skeletal muscle, ileum (with Peyer's patches), jejunum, lungs, lymph nodes (mesenteric and mandibular), sciatic nerves, pancreas, pituitary glands, rectum, salivary glands, skin (with mammary glands), spinal cord (cervical, thoracic, and lumbar), sternum, stomach, tail (identification mark), thyroid (with parathyroid glands), trachea, urinary bladder, and vagina.

##### 90‐day repeated dose toxicity

Potential toxicity of LI12542F6 following administration for 90 days was evaluated according to OECD Guideline 408 (1998). Twenty Wistar rats (10M + 10F) (Vivo Bio Tech Ltd, Telangana, India) were used in each of 4 main groups including a vehicle control (G1) and low, mid, and high treatment groups (G2 1,000 mg/kg; G3 1,500 mg/kg; and G4 2,000 mg/kg). Ten rats (5M + 5F) were used in each of the 2 recovery groups (G1R control and G4R high dose). The main groups were treated for 90 days and the recovery groups continued on test without treatment for an additional 28 days. The vehicle for the test material was 0.5% (w/v) carboxy methyl cellulose sodium salt. Doses were administered by gavage (10 ml/kg bw). Dose verification and homogeneity were analyzed using HPLC by the laboratory on days 1, 42, and 87, and concentrations were found to be within the acceptable limits.

Animals were observed twice daily for morbidity and mortality, and cage side evaluations of general clinical signs were made daily. Examination of detailed clinical signs was made pretreatment and after each week of the study. Ophthalmic examinations were performed before treatment commenced, at the end of treatment for main groups, and at the end of the recovery period for recovery groups. The functional observational battery was performed before treatment started (day 0), and during the 13^th^ week (days 85–91) (main groups) and 17^th^ week (days 113–119) (recovery groups). Observations were recorded for animals in their home cages, when removed and handled, and in open field, to note any abnormal activity or features. Functional tests, sensory reactivity, landing foot splay, grip strength performance, and motor activity were measured. Body weight and food consumption were noted weekly. Clinical pathology examinations took place on day 91 for the main groups or day 119 for the recovery groups. Blood was collected after overnight fasting (water ad libitum) for hematology and clinical chemistry analysis at the end of the study (days 91 and 119 for main and recovery groups, respectively). Following sacrifice, all animals were subjected to gross pathological examination, and then removal of organs for weighing and histopathology. The hematological and clinical chemistry parameters, as well as the tissues and organs for weighing and histopathological examination, were the same as for the 28‐day study.

#### 2.4.4 Statistical analysis

The study data were subjected to statistical analyses using GraphPad Prism software. All data were checked for normality and homogeneity prior to statistical comparisons. All normal and homogenous data were analyzed using one‐way ANOVA followed by Dunnett's multiple comparisons in main groups and Student's *t* test in recovery groups, whereas non‐normal and/or nonhomogeneous data were analyzed using Kruskal–Wallis test followed by Dunnett's multiple comparisons in main groups and Mann–Whitney *U* test in recovery groups, respectively. All analyses and comparisons were evaluated at the 95% level of confidence (*p* < 0.05).

## 3 RESULTS

### 3.1 Ames test

Preliminary toxicity determination on TA100 strain found slight reduction in revertants/plate and in the background lawn at the highest dose of 5,000 μg/plate (See Supporting information Table S1). Results from triplicate cultures in the initial mutation assay on the panel of bacterial strains using the plate incorporation test (Tables 1 and 2) and in the confirmatory test using the preincubation assay (See Supporting information Tables S2 and S3) showed that LI12542F6 did not induce a dose‐dependent increase in revertant colonies at doses of up to and including 5,000 μg/plate, with and without metabolic activation (S9). Slight cytotoxicity was found at the highest dose tested. All negative and positive control values were within expected and normal ranges. Thus, it is concluded that LI12542F6 is not mutagenic up to 5,000 μg/plate in the Ames test.

**Table 1:** Observation on initial mutation assay evaluating genotoxicity potential of LI12542F6 in the presence of metabolic activation

Treatment (µg/plate)	No. of revertants/plate									
	TA98		TA100		TA1535		TA1537		WP2*uvrA*(pkm101)	
	Mean ± SD	Ratio^a^	Mean ± SD	Ratio^a^	Mean ± SD	Ratio^a^	Mean ± SD	Ratio^a^	Mean ± SD	Ratio^a^
Vehicle control DMSO	26 ± 2	NA	88 ± 3	NA	13 ± 2	NA	11 ± 2	NA	135 ± 3	NA
50	23 ± 4	0.88	83 ± 2	0.94	10 ± 2	0.77	9 ± 3	0.82	129 ± 3	0.96
158	22 ± 1	0.85	73 ± 3	0.83	12 ± 1	0.92	7 ± 1	0.64	131 ± 3	0.97
500	22 ± 3	0.85	69 ± 4	0.78	8 ± 2	0.62	8 ± 2	0.73	126 ± 2	0.93
1581	18 ± 3	0.69	68 ± 2	0.77	8 ± 2	0.62	7 ± 2	0.64	126 ± 4	0.93
5000	15 ± 2	0.58	46 ± 3	0.52	5 ± 2	0.38	5 ± 1	0.45	113 ± 4	0.84
Positive control	569^b^± 15	21.88^b^	883^b^ ± 7	10.03^b^	143^b^ ± 8	11.00 b	149^b^ ± 12	13.55^b^	591^c^ ± 6	4.38^c^

NA: not applicable; SD: standard deviation.

^a^Ratio of treated/vehicle control (mean revertants per plate). ^b^TA98, TA100, TA1535, TA1537: 2‐Aminoanthracene (4 µg/plate). ^c^WP2*uvrA*(pkm101): 2‐Aminoanthracene (30 µg/plate).

**Table 2:** Observation on initial mutation assay evaluating genotoxicity potential of LI12542F6 in the absence of metabolic activation

Treatment (µg/plate)	No. of revertants/plate									
	TA98		TA100		TA1535		TA1537		WP2*uvrA*(pkm101)	
	Mean ± SD	Ratio^a^	Mean ± SD	Ratio^a^	Mean ± SD	Ratio^a^	Mean ± SD	Ratio^a^	Mean ± SD	Ratio^a^
Vehicle control DMSO	24 ± 2	NA	90 ± 3	NA	11 ± 2	NA	11 ± 3	NA	134 ± 4	NA
50	23 ± 3	0.96	84 ± 2	0.93	9 ± 2	0.82	9 ± 2	0.82	127 ± 2	0.95
158	21 ± 3	0.88	75 ± 3	0.83	10 ± 3	0.91	8 ± 2	0.73	127 ± 5	0.95
500	22 ± 3	0.92	65 ± 3	0.72	9 ± 2	0.82	9 ± 1	0.82	125 ± 4	0.93
1581	18 ± 3	0.75	65 ± 4	0.72	10 ± 3	0.91	8 ± 2	0.73	124 ± 3	0.93
5000	14 ± 2	0.58	44 ± 3	0.49	5 ± 2	0.45	5 ± 1	0.45	112 ± 3	0.84
Positive control	275^b^ ± 13	11.46 b	857^c^ ± 8	9.52 c	141^c^ ± 8	12.82^c^	145^d^ ± 13	13.18 d	564^e^ ± 10	4.21^e^

NA: not applicable; SD: standard deviation.

^a^Ratio of treated/vehicle control (mean revertants per plate); ^b^TA98: 2‐Nitrofluorene (2 µg/plate); ^c^TA100, TA 1535: Sodium azide (1 µg/plate); ^d^TA1537: 9‐Aminoacridine (50 µg/plate); ^e^WP2*uvrA*(pkm101): 4‐Nitroquinoline‐N‐oxide (4 µg/plate).

### 3.2 In vivo mouse bone marrow micronucleus test

No dose of LI12542F6 or the positive control cyclophosphamide showed cytotoxicity as determined by a lack of reduction in the PCE‐to‐total erythrocyte (TE) ratio compared to the vehicle control (Table 3). In comparison with the vehicle control group, LI12542F6 did not induce an increase in the frequency of micronucleated PCE (MNPCE), whereas the positive control showed a significant increase (Table 3). The conclusion is that LI12542F6 does not induce micronuclei in the mouse.

**Table 3:** Effect of LI12542F6 on micronucleus frequency in mouse bone marrow erythrocytes

Test material	Dose (mg/kg B.Wt)	Mean PCE:TE	% Reduction	% MNPCE ± SD
Vehicle control	0	0.609	NA	0.11 ± 0.04
LI12542F6	500	0.620	−1.8	0.09 ± 0.08
LI12542F6	1,000	0.609	−0.1	0.07 ± 0.04
LI12542F6	2,000	0.606	0.4	0.05 ± 0.03
Cyclophosphamide monohydrate	40	0.614	−0.9	2.03 ± 0.10***

Values are represented as mean ± SD; *n* = 5; ***p < 0.0001, comparison between treatment and control groups in one‐way ANOVA. PCEs: polychromatic erythrocytes; TE: total erythrocyte; MNPCE: micronucleated polychromatic erythrocyte; SD: standard deviation.

### 3.3 Acute oral toxicity study (data not shown)

LI12542F6 did not induce any mortality, morbidity, or clinical signs at the limit dose of 2,000 mg/kg bw in any of the five test animals at 30 min, 1, 2, 3, and 4 hr, and thereafter daily until day 14 postdosing. Each animal was subjected to gross necropsy, and no abnormality was found except for spleen enlargement in one animal. Upon histopathological examination, moderate extramedullary hematopoiesis (erythroid type) was found. This finding is known to be spontaneous in the female rat (Boorman, Eustis, & Elwell, 1990) and is considered unrelated to treatment. The oral LD_50_ was reported to be greater than 2,000 mg/kg bw.

### 3.4 Acute dermal toxicity study

LI12542F6 did not induce any mortality, morbidity, clinical signs, or gross pathological findings at the limit dose of 2,000 mg/kg bw in any of the five test animals (data not shown). This observation suggests that the dermal LD_50_ of LI12542F6 is greater than 2,000 mg/kg bw.

### 3.5 Acute dermal irritation/corrosion study

The test material did not induce any mortality, morbidity, clinical signs, or gross pathological findings. The mean irritation score for erythema (reddening of the skin) for animals at 24, 48, and 72 hr following test material application was ≤1.67, findings that were reversible in the 14‐day recovery period (data not shown). The classification of LI12542F6, therefore, is a “mild irritant” (Category 3 with a mean value ≥1.5 and <2.3), according to the GHS Harmonized Integrated Classification System (2015).

### 3.6 Acute eye irritation/corrosion study

The test material did not induce any mortality, morbidity, clinical signs, or gross pathological findings. Lacrimation occurred in all three animals, and all animals showed weight gains. Animal 1 demonstrated conjunctival redness at 1 hr that persisted until 72 hr with chemosis observed at 1 hr at 24, 48, and 72 hr, observations that subsided by day 7. No opacity was found in animal 1, and the iris was normal in all 3 test animals. Animals 2 and 3 showed conjunctival redness, chemosis, and corneal opacity through day 14, and the effects on the eye were reversible by day 21. Results are shown in Table 4 for the left eye (treated). No signs of irritation, lacrimation, swelling, or redness were observed in the untreated right eye, considered as the control (data not shown). Based on the scores for corneal opacity and chemosis, the test material is classified “Category 2A (irritating to eyes – reversible eye effect)” (GHS, 2015).

**Table 4:** Effect of LI12542F6 on eye irritation scores (left eye) in New Zealand white rabbit

Eye reactions	Time interval							
	PE	1 hr	24 hr	48 hr	72 hr	Day 7	Day 14	Day 21
Opacity								
No ulceration or opacity	3/3*	3/3	1/3	1/3	1/3	1/3	–	2/2
Easily discernible translucent area; details of iris slightly obscured	–	–	2/3	2/3	2/3	2/3	2/2	–
Area of corneal opacity								
Zero	3/3	3/3	1/3	1/3	1/3	1/3	–	2/2
Greater than one‐quarter, but less than half	–	–	2/3	2/3	2/3	2/3	2/2	–
Iris								
Normal	3/3	3/3	3/3	3/3	3/3	3/3	2/2	2/2
Conjunctivae								
Blood vessels normal	3/3	–	–	–	–	1/3	–	2/2
Some blood vessels definitely hyperemic	–	3/3	3/3	3/3	3/3	2/3	2/2	–
Chemosis								
No swelling (normal)	3/3	–	–	–	–	1/3	–	2/2
Some swelling above normal	–	–	1/3	1/3	2/3	2/3	2/2	–
Swelling with partial eversion of lids	–	1/3	–	2/3	1/3	–	–	–
Swelling with lids about half closed	–	1/3	2/3	–	–	–	–	–
Swelling with lids more than half closed	–	1/3	–	–	–	–	–	–

PE: pre‐exposure; hr: hour. *Number of animals showing eye reactions out of total number of animals scored.

### 3.7 28‐day repeat dose toxicity study

No morbidity, mortality, cage side or detailed clinical signs, or ocular lesions associated with the administration of the test material were evident. Random increases in body weight (G2 male) and in net body weight gain (G3 female), and decreases in body weight (G4R male) and in net body weight gain (week 1, G4R male rats) were not dose related and considered not to be toxicologically significant. Similarly, sporadic decreases in feed consumption, changes in hematological and clinical chemistry parameters, variations in absolute and relative organ weights, and gross pathological abnormalities were few in number, not dose related, and considered to be irrelevant to the treatment (data not shown).

Histopathologic results are summarized in Table 5a and b. Treatment‐related histopathologic findings were confined to minimal to moderate hyperplasia/hyperkeratosis of the epithelial mucosa and minimal to mild inflammation in the associated submucosa of the nonglandular forestomach of mid‐ and high‐dose groups of LI12542F6 in males and females (Table 5a and b, respectively). Severity and frequency of these effects were lower in the high‐dose recovery group, with evidence of significant recovery (presence of angiogenesis along with fibroplasia) in the absence of treatment. These findings are relatively common in rats subjected to repeated gavage, as the forestomach serves as a storage organ and is subject to local irritation and inflammation. Given that the human does not have a forestomach and there was no system toxicity in other organs, this finding is not considered relevant to human risk assessment (Proctor et al., 2007).

**Table 5:** Effects of graded doses of LI12542F6 on histopathological findings in (a) male and (b) female Wistar rats in 28‐day repeated dose toxicity study

Organs/findings	Severity	Incidence of findings					
		Control	500 mg/kg BW	1000 mg/kg BW	1500 mg/kg BW	Control reversal	High‐dose reversal
(a)							
Liver							
Infiltration, MNC, hepatocellular	Minimal	1/5^a^	NE	NE	1/5	–	–
Lungs							
Foamy macrophages, alveolar	Marked	1/5	0/5	0/5	0/5	–	–
Hemorrhage, alveolar (gross lesion)	Mild	0/5	1/5	NE	0/5	–	–
Congestion, alveolar (gross lesion)	Minimal	–	–	–	–	1/5	NE
Lymph node (mesenteric and mandibular)							
Histiocytosis/erythrocytosis, sinus	Minimal	0/5	NE	NE	1/5	–	–
Increased number/density, lymphocytes, paracortex	Minimal	1/5	NE	NE	0/5	–	–
Plasmacytosis, sinus	Minimal	0/5	NE	NE	1/5	–	–
Pancreas							
Degeneration/atrophy, acinar	Minimal	0/5	NE	NE	1/5	–	–
Stomach (nonglandular)							
Epithelial hyperplasia/hyperkeratosis	Minimal	0/5	0/5	4/5	1/5	1/5	2/5
	Mild	0/5	0/5	1/5	2/5	–	–
	Moderate	0/5	0/5	0/5	2/5	–	–
Infiltration, MNC/PMNC, submucosa	Minimal	0/5	0/5	0/5	1/5	0/5	1/5
	Mild	0/5	0/5	0/5	1/5	–	–
Angiogenesis/fibroplasia, submucosa	Mild	0/5	0/5	0/5	1/5	–	–
	Minimal	–	–	–	–	0/5	3/5
Epididymides							
Oligospermia	–	1/5	NE	NE	0/5	–	–
Spermatic granuloma	Moderate	1/5	NE	NE	0/5	–	–
Infiltration, MNC, interstitial	Minimal	0/5	NE	NE	1/5	–	–
	Moderate	1/5	NE	NE	0/5	–	–
Prostate							
Infiltration, MNC, interstitial	Minimal	1/5	NE	NE	1/5	–	–
(b)							
Adrenals							
Cortical tissue, accessory	–	1/5^a^	NE	NE	2/5	–	–
Ileum with Peyer's patches							
Increased size/density, lymphocytes, GALT	Mild	1/5	NE	NE	0/5	–	–
Eyes							
Rosettes, retina	Minimal	0/5	NE	NE	1/5	–	–
Heart							
Infiltration, MNC, myometrium	Minimal	1/5	NE	NE	0/5	–	–
Kidneys							
Epithelial hyperplasia/MNC infiltration, pelvis	Mild	0/5	NE	NE	1/5	–	–
Liver							
Infiltration, MNC, portal	Minimal	1/5	NE	NE	1/5	–	–
Lungs							
Foamy macrophages, alveolar	Moderate	1/5	NE	NE	0/5	–	–
Infiltration, MNC, perivascular	Minimal	0/5	NE	NE	1/5	–	–
Lymph node (mandibular)							
Erythrocytosis, sinus	Mild	1/5	NE	NE	0/5	–	–
Pancreas							
Degeneration/atrophy, acinar	Minimal	1/5	NE	NE	0/5	–	–
Pituitary gland							
Pseudocyst, pars distalis/intermedia	Minimal	2/5	NE	NE	0/5	–	–
Stomach (nonglandular)							
Epithelial hyperplasia/hyperkeratosis	Minimal	0/5	0/5	2/5	1/5	0/5	2/5
	Mild	0/5	0/5	2/5	1/5	–	–
	Moderate	0/5	0/5	0/5	3/5	–	–
Infiltration, MNC/PMNC, submucosa	Minimal	0/5	0/5	1/5	1/5	0/5	2/5
	Mild	0/5	0/5	1/5	2/5	–	–
Angiogenesis/fibroplasia, submucosa	Minimal	0/5	0/5	0/5	1/5	0/5	1/5
	Mild	0/5	0/5	0/5	2/5	0/5	1/5
Squamous cyst	Minimal	–	–	–	–	0/5	1/5

“–” indicates not applicable; NE: not examined; MNC: mononuclear cells; PMNC: polymorphonuclear cells; GALT: gut‐associated lymphoid tissue.

a Number of animals showing findings out of total number of animals in the group, *n* = 5.

### 3.8 90‐day repeated dose toxicity study

No morbidity, mortality, cage side or detailed clinical signs, or ocular lesions were found in any rats in this study. Functional observational battery (FOB) tests were normal for all groups at pre‐ and posttreatment. No abnormalities in behavior, activity, or functionality were observed in home cages, during removal from cages, or in open field tests. No treatment‐related abnormalities were found during tests of sensory reactivity, grip strength, motor activity, or landing foot splay (data not shown).

Although significant changes in body weight (Table 6a and b) and food intake (Table 7a and b) were found at various times in male rats, they were not treatment related. Males in the mid‐dose group (1500 mg/kg; G3) showed statistically significant, but not dose‐dependent, decreases in mean body weight at days 49, 77, 84, and 91 (Table 6a). During the course of study, analyses of time‐dependent body weight changes reveal that the natural weight gain was significantly reduced in male animals at mid‐dose (G3) on day 49 and days 77–91. These inconsistent changes are not considered to be toxicologically relevant or treatment related as they were isolated incidents with no accompanying observations in histopathology. Significant reductions in feed consumption were found in G3 males on days 49, 56, and 77 and in G4 males on days 7 and 35 (Table 7a). Significant increases occurred in G4R males on days 28 and 119 (Table 7a), in G2 females on day 70, and in G4 females on day 28 (Table 7b). These random changes in feed intake are considered not to be toxicologically relevant or related to treatment. Consistently high feed consumption in G4R (high‐dose recovery group) females from day 14–119 does not appear to be treatment‐related; is not replicated in the G4 high‐dose group; may in part be due to the fact that feed consumption in the G1R control group is consistently low compared to the G1 control for most (84 days) of the duration of treatment (Table 7b); and in any event would not likely be considered to be an adverse outcome.

**Table 6:** Effect of 90‐day oral administration of LI12542F6 on body weights of (a) male and (b) female Wistar rats. Effect of 90‐day oral administration of LI12542F6 on body weights of female Wistar rats

Body weight (g)						
Day	Male					
	Main groups				Reversal groups	
	G1 Vehicle control	G2 LI12542F6‐1000 mg/kg BW	G3 LI12542F6‐1500 mg/kg BW	G4 LI12542F6‐2000 mg/kg BW	G1R Vehicle control	G4R LI12542F6‐2000 mg/kg BW
(a)						
0	181.50 ± 15.93	181.06 ± 16.85	176.85 ± 14.82	178.75 ± 14.48	173.17 ± 7.91	178.50 ± 8.71
7	215.31 ± 22.99	211.85 ± 24.15	200.87 ± 19.08	205.86 ± 19.22	209.38 ± 9.61	215.49 ± 16.41
14	250.74 ± 31.76	238.23 ± 28.65	226.95 ± 20.95	233.85 ± 21.58	238.82 ± 14.00	250.92 ± 23.99
21	277.14 ± 38.39	260.46 ± 33.83	247.71 ± 24.67	257.95 ± 27.10	260.77 ± 18.01	276.93 ± 27.04
28	301.50 ± 44.65	281.75 ± 37.81	265.61 ± 28.98	275.44 ± 30.03	282.69 ± 21.74	299.03 ± 29.20
35	317.01 ± 46.23	296.60 ± 40.64	279.04 ± 29.92	289.28 ± 33.47	297.16 ± 25.33	313.75 ± 33.87
42	329.71 ± 46.90	307.05 ± 40.15	290.62 ± 31.68	301.84 ± 35.06	312.07 ± 28.36	325.18 ± 37.45
49	349.11 ± 56.75	320.06 ± 41.84	300.80 ± 33.67*↓	313.12 ± 38.46	323.56 ± 27.50	338.60 ± 39.39
56	353.34 ± 51.49	328.90 ± 42.98	309.87 ± 31.85	321.72 ± 37.99	330.81 ± 28.92	350.07 ± 43.01
63	364.60 ± 53.03	336.50 ± 41.91	318.41 ± 34.05	332.12 ± 39.80	343.11 ± 31.65	361.65 ± 46.87
70	372.17 ± 54.66	344.10 ± 43.64	325.90 ± 34.00	340.78 ± 42.27	350.80 ± 29.71	370.57 ± 49.28
77	380.28 ± 55.22	349.00 ± 43.78	330.29 ± 35.63*↓	345.23 ± 43.01	356.75 ± 31.71	375.29 ± 48.86
84	387.89 ± 56.77	356.05 ± 42.58	334.95 ± 36.49*↓	351.27 ± 43.55	363.21 ± 32.10	385.85 ± 50.38
91	391.20 ± 54.93	359.50 ± 43.12	336.20 ± 36.40*↓	351.10 ± 44.99	369.05 ± 32.29	387.55 ± 50.59
98					371.59 ± 31.46	393.11 ± 52.40
105					375.49 ± 33.28	399.96 ± 55.41
112					377.05 ± 33.04	404.18 ± 56.10
119					376.80 ± 33.88	404.69 ± 56.25

Body weight (g)						
Day	Female					
	Main groups				Reversal groups	
	G1 Vehicle control	G2 LI12542F6‐1000 mg/kg BW	G3 LI12542F6‐1500 mg/kg BW	G4 LI12542F6‐2000 mg/kg BW	G1R Vehicle control	G4R LI12542F6‐2000 mg/kg BW
(b)						
0	147.96 ± 12.88	150.97 ± 13.48	148.94 ± 13.63	147.37 ± 12.61	145.67 ± 9.14	142.65 ± 10.10
7	160.11 ± 11.26	166.69 ± 14.55	163.24 ± 11.29	162.35 ± 13.96	160.79 ± 9.03	159.29 ± 11.22
14	175.09 ± 13.85	178.54 ± 13.36	175.39 ± 10.04	173.75 ± 16.61	169.58 ± 10.57	173.63 ± 12.36
21	183.41 ± 14.54	187.92 ± 12.95	186.05 ± 11.21	183.27 ± 15.49	179.54 ± 7.15	184.89 ± 12.37
28	191.20 ± 17.80	197.08 ± 12.18	194.43 ± 13.55	191.50 ± 16.89	187.41 ± 6.80	193.30 ± 13.47
35	195.31 ± 17.72	201.18 ± 12.29	200.91 ± 14.27	195.47 ± 16.61	191.03 ± 7.76	199.89 ± 16.68
42	199.59 ± 17.17	205.12 ± 11.24	206.80 ± 13.60	200.25 ± 17.64	194.86 ± 8.97	203.42 ± 17.14
49	203.94 ± 19.06	209.07 ± 10.98	210.44 ± 14.73	202.99 ± 18.21	196.84 ± 8.96	208.58 ± 17.17
56	207.36 ± 19.71	211.76 ± 11.74	213.71 ± 15.66	206.59 ± 19.69	201.56 ± 7.48	211.53 ± 17.06
63	208.46 ± 19.35	214.44 ± 12.30	216.14 ± 16.22	209.41 ± 20.10	206.77 ± 9.31	214.38 ± 17.98
70	210.28 ± 19.57	217.97 ± 12.27	218.50 ± 16.59	211.48 ± 20.69	207.79 ± 9.52	218.69 ± 17.71
77	212.69 ± 19.51	220.44 ± 11.10	220.14 ± 16.35	212.47 ± 21.18	209.40 ± 8.57	220.6 ± 17.25
84	214.93 ± 20.41	222.84 ± 11.57	222.80 ± 17.61	214.36 ± 21.79	212.75 ± 9.42	222.63 ± 17.75
91	215.77 ± 20.78	222.25 ± 11.37	221.84 ± 16.94	213.49 ± 21.01	215.81 ± 11.19	224.57 ± 19.05
98					215.63 ± 10.49	224.48 ± 19.45
105					213.94 ± 8.55	226.27 ± 19.24
112					217.22 ± 8.38	227.15 ± 18.08
119					217.38 ± 8.10	226.81 ± 18.45

*Notes*

(a) *n* = 10 in main groups and *n* = 5 in reversal groups; *↓: Significantly lower than the control group, *p* < 0.05. (b) *n* = 10 in main groups and *n* = 5 in reversal groups.

**Table 7:** Weekly feed consumption by LI12542F6 gavaged (a) male and (b) female Wistar rats

Feed consumption (g)						
Days	Male					
	Main groups				Recovery groups	
	G1 Vehicle control	G2 LI12542F6‐1000 mg/kg BW	G3 LI12542F6‐1500 mg/kg BW	G4 LI12542F6‐2000 mg/kg BW	G1R Vehicle control	G4R LI12542F6‐2000 mg/kg BW
(a)						
0–7	132.49 ± 9.63	124.23 ± 10.91	124.85 ± 4.93	117.34 ±6.32**↓	135.49 ± 8.29	134.25 ± 0.48
8–14	142.31 ± 10.95	134.08 ± 16.83	131.94 ± 14.82	126.51 ± 3.76	138.43 ± 8.96	142.81 ± 10.04
15–21	145.65 ± 11.86	136.59 ± 18.85	132.36 ± 11.41	132.88 ± 7.15	143.09 ± 9.25	148.61 ± 3.90
22–28	144.79 ± 13.99	143.14 ± 21.84	133.03 ± 10.95	130.87 ± 4.08	143.18 ± 6.87	154.21 ± 0.31**↑
29–35	145.06 ± 7.44	140.86 ± 18.47	130.97 ± 10.40	127.71 ± 2.55*↓	141.07 ± 5.29	146.63 ± 12.70
36–42	132.11 ± 6.37	134.74 ± 15.14	126.24 ± 8.07	128.59 ± 1.34	144.15 ± 5.11	137.72 ± 10.22
43–49	134.31 ± 6.94	134.65 ± 16.10	121.02 ± 6.36**↓	128.94 ± 1.49	140.20 ± 2.81	139.19 ± 10.67
50–56	132.69 ± 6.11	132.94 ± 15.10	123.18 ± 5.79*↓	125.38 ± 1.03	137.31 ± 1.68	136.90 ± 10.34
57–63	131.85 ± 5.10	127.11 ± 11.54	125.09 ± 9.34	124.45 ± 1.78	133.85 ± 5.14	137.63 ± 9.78
64–70	129.63 ± 5.83	126.09 ± 7.28	126.56 ± 8.28	121.19 ± 9.34	132.89 ± 0.41	139.25 ± 6.74
71–77	142.16 ± 15.52	128.48 ± 12.48	117.69 ± 8.13***↓	127.14 ± 3.23	132.29 ± 3.22	137.96 ± 9.08
78–84	127.30 ± 1.90	126.58 ± 14.67	123.40 ± 12.23	125.06 ± 2.90	128.51 ± 2.12	133.98 ± 8.22
85–91	89.20 ± 6.38	92.21 ± 10.31	85.80 ± 4.08	87.87 ± 2.65	129.83 ± 1.81	131.07 ± 8.87
92–98					131.35 ± 4.78	137.06 ± 13.03
99–105					126.92 ± 0.29	139.26 ± 15.19
106–112					125.20 ± 0.91	137.88 ± 11.65
113–119					89.64 ± 0.02	101.24 ± 10.46**↑

Feed consumption (g)						
Days	Female					
	Main groups				Recovery groups	
	G1 Vehicle control	G2 LI12542F6‐1000 mg/kg BW	G3 LI12542F6‐1500 mg/kg BW	G4 LI12542F6‐2000 mg/kg BW	G1R Vehicle control	G4R LI12542F6‐2000 mg/kg BW
(b)						
0–7	96.99 ± 8.92	88.97 ± 15.64	89.19 ± 3.61	86.31 ± 5.23	78.61 ± 9.15	83.62 ± 22.75
8–14	98.02 ± 7.35	98.17 ± 2.04	93.29 ± 6.88	94.35 ± 5.27	91.06 ± 2.53	102.61 ± 1.07**↑
15–21	95.96 ± 9.64	99.12 ± 1.75	96.15 ± 5.56	96.48 ± 2.55	90.90 ± 0.66	102.00 ± 3.36**↑
22–28	95.01 ± 10.56	86.75 ± 14.56	98.53 ± 8.35	109.29 ± 14.35*↑	90.88 ± 0.37	101.94 ± 3.79**↑
29–35	97.53 ± 12.23	99.37 ± 5.86	96.09 ± 5.95	87.14 ± 15.05	87.15 ± 1.80	100.11 ± 3.93**↑
36–42	94.37 ± 9.63	95.19 ± 2.67	95.14 ± 6.01	90.37 ± 5.51	88.84 ± 1.55	97.39 ± 0.74**↑
43–49	93.73 ± 11.01	97.67 ± 2.55	96.03 ± 7.57	90.39 ± 5.41	88.48 ± 2.35	95.64 ± 1.71**↑
50–56	91.89 ± 7.55	95.07 ± 1.99	92.73 ± 8.13	90.25 ± 5.56	87.23 ± 2.45	98.96 ± 4.93**↑
57–63	89.26 ± 8.00	93.08 ± 1.42	91.98 ± 7.06	87.60 ± 6.97	85.51 ± 0.75	96.15 ± 2.31**↑
64–70	90.92 ± 10.28	102.07 ± 7.30**↑	91.05 ± 8.62	90.32 ± 4.59	80.71 ± 10.72	96.26 ± 1.78**↑
71–77	92.00 ± 9.04	92.37 ± 2.13	88.54 ± 8.22	85.19 ± 6.50	85.88 ± 1.97	95.57 ± 2.55**↑
78–84	88.12 ± 9.73	90.43 ± 1.52	85.39 ± 6.70	83.81 ± 5.24	86.14 ± 0.05	94.04 ± 3.40**↑
85–91	62.63 ± 6.28	64.96 ± 2.38	61.34 ± 3.70	60.54 ± 2.82	83.05 ± 3.29	94.13 ± 3.17**↑
92–98					79.15 ± 8.45	92.94 ± 1.25**↑
99–105					79.90 ± 7.02	94.61 ± 5.33**↑
106–112					80.27 ± 7.15	93.36 ± 2.12**↑
113–119					53.81 ± 5.19	67.13 ± 2.31**↑

*Notes*

(a) *n* = 10 in main groups and *n* = 5 in reversal groups;*↓: Significantly lower than the control group, *p* < 0.05. **↓: Significantly lower than the control group, *p* < 0.01. ***↓: Significantly lower than the control group, *p* < 0.001**↑: Significantly higher than the control group, *p* < 0.01. (b) *n* = 10 in main groups and *n* = 5 in reversal groups;*↑: Significantly higher than the control group, *p* < 0.05. **↑: Significantly higher than the control group, *p* < 0.01.

Although sporadic mean values were statistically significant for various hematological parameters (Supporting information Table 4a,b), they were within the normal range for the species and are considered to be unrelated to treatment. Increases in platelet count were observed in low‐dose (G2) female and mid‐dose (G3) male groups; in low‐dose (G2) female animals, decreases in hemoglobin and hematocrit were found; and, in high‐dose recovery (G4R) males, increases in hemoglobin, hematocrit, and mean corpuscular volume were observed that were not found in the G4 group.

Several statistically significant clinical chemistry findings (Supporting information Table 5a,b) were found. In G2 and G3 male rats, decreases in total bilirubin and increases in sodium levels were observed; in G4 males, a reduced aspartate aminotransferase (AST) value was found. Non‐dose‐related decreases in AST levels in G2, G3, and G4 females were within the range of historical controls and are considered to have no toxicological significance. Additional sporadic findings include reduced alanine aminotransferase level in G4 females, increased sodium level G3 females, increased glucose and decrease in calcium in G4R females.

There were no statistically significant changes in absolute organ weights (Table 5a and b) in any of the male or female G2, G3, or G4 treatment groups; G4R males showed significant increases in absolute kidney and thymus weights and in relative liver and thymus weights. In male and female rats, a statistically significant increase in the relative organ weight of liver was observed in the mid‐dose groups. In female rats, increased relative organ weight of liver in the mid‐dose group (G3) and increased relative spleen weight in the high‐dose group (G4) were observed. These findings did not exhibit dose dependency, do not correspond with any other pathological findings, and are considered to have no toxicological relevance. No changes in organ weights (Supporting information Table 6a,b), relative organ weights, or gross pathological findings were treatment related in either sex (data not shown). The findings noted above to be irrelevant in the forestomach of animals in the 28‐day study were not found in the 90‐day study, confirming that these observations were transitory side effects from repeated gavage in the earlier study. Various minimal to mild, not statistically significant histopathological findings were noted in G1 and G4 groups (Table 8a and b) and are considered to be unrelated to treatment. Based on these results, the no observed adverse effect level (NOAEL) of LI12542F6 in this 90‐day study is greater than 2,000 mg/kg bw, the highest dose tested.

**Table 8:** Histopathological findings—(a) male and (b) female (90‐day repeated dose toxicity study)

Organs/findings	Severity/presence	Incidence of findings^a^	
		Vehicle control	LI12542F6‐2000 mg/kg BW
(a)			
Colon			
Mononuclear cell infiltration, submucosa, focal	Minimal	1/10	0/10
Gut‐associated lymphoid tissue	Present	0/10	1/10
Duodenum			
Gut‐associated lymphoid tissue	Present	2/10	1/10
Jejunum			
Gut‐associated lymphoid tissue	Present	1/10	2/10
Liver			
Mononuclear cells, focal/multifocal	Minimal	5/10	0/10
Pituitary glands			
Cyst, pars intermedia	Present	0/10	1/10
Cyst, pars distalis	Present	0/10	1/10
Urinary bladder			
Eosinophilic material, lumen	Minimal	0/10	1/10
	Mild	0/10	2/10
	Marked	6/10	2/10
Prostate gland			
Mononuclear cell infiltrate, interstitial, focal/multifocal	Minimal	4/10	0/10
	Mild	1/10	0/10
(b)			
Adrenals glands			
Accessory cortical nodule	Present	0/10	1/10
Colon			
Mononuclear cell infiltration, submucosa, multifocal	Mild	0/10	1/10
Liver			
Mononuclear cell, focal	Minimal	2/10	4/10
Lungs			
Mononuclear cell infiltration, focal	Mild	0/10	1/10
Pituitary glands			
Rathke's cleft (persistent)	Present	1/10	0/10
Small cyst in pars nervosa	Present	0/10	1/10
Small cyst	Present	1/10	0/10
Thyroid with parathyroid glands			
Mononuclear cell infiltration	Minimal	1/10	0/10
Spinal cord			
Cyst	Present	1/10	0/10
Thymus	–	0/10	0/10
Small cyst	Present	0/10	2/10
Urinary bladder			
Mononuclear cell infiltration mucosa, focal	Minimal	0/10	1/10

a Number of animals showing findings out of total number of animals in the group, *n* = 10.

## 4 DISCUSSION

Various parts of *S. indicus* and *M. indica* plants have been used for thousands of years for a wide variety of ethnomedicinal purposes (George et al., 2017; Masud Parvez, 2016; Ramachandran, 2013; Sharma et al., 2001). A number of studies with findings similar to those found with LI12542F6 have been published on mango stem bark extract (MSBE) as well as on mangiferin, the main active component of MSBE, and on *S. indicus* extracts. Rodeiro et al. (2006) performed an extensive battery of genotoxicity tests on MSBE including the *Salmonella typhimurium* bacterial reversion test and the in vivo mouse micronucleus test that were similarly negative as for LI12542F6. In addition, Rodeiro et al. (2006) found that the Cuban product Vimang^®^, containing MSBE, was negative in the in vitro micronucleus and in an in vivo comet assay. Subsequently, Rodeiro et al. (2012) tested mangiferin in another battery of genotoxicity tests. As found for Vimang^®^ (Rodeiro et al., 2006) and for LI12542F6, mangiferin was negative in the Ames *Salmonella* and in the mouse micronucleus tests. In addition, Rodeiro et al. (2012) found that mangiferin did not induce DNA damage in a bacterial SOS chromotest or in an in vivo comet assay. Saiyed et al. (2015) tested Meratrim^®^, a 3:1 mixture of *S. indicus* paste and *Garcinia mangostana* powder (with 55% excipients), in a battery of genotoxicity tests and rodent toxicity studies. Similar to results for LI12542F6, Meratrim^®^ did not induce genotoxicity in the Ames bacterial mutation test or in the mouse micronucleus assay. Furthermore, Meratrim^®^ did not induce chromosome aberrations in cultured lymphocytes. Together, these results for LI12542F6 itself, as well as for its constituents *S. indicus* and *M. indica* in various formulations, demonstrate a lack of genotoxic potential for LI12542F6.

Also in agreement with reported observations on Vimang^®^ (Garrido et al., 2009), the present findings on LI12542F6 showed no lethality in oral and dermal LD_50_ limit tests in mice and rats (at 2,000 mg/kg body weight), with no adverse effects. Similarly, Nahata and Dixit (Nahata & Dixit, 2011) and Ambikar and Mohanta (Ambikar & Mohanta, 2013) found that extracts of *S. indicus* showed LD_50_ values greater than 2,000 mg/kg body weight with no observed adverse effects. Meratrim^®^ was found to have an oral LD_50_ in rats in excess of 5,000 mg/kg body weight, with a few minor effects in different animals, a dermal LD_50_ greater than 2,000 mg/kg with no adverse effects seen, no dermal irritation and slight eye irritation (Saiyed et al., 2015). A NOAEL for this combination ingredient that included *S. indicus* was concluded to be 1000 mg/kg of body weight/day in male and female SD rats, which was the highest dose tested. Although the herbal blend LI12542F6 containing *S. indicus* and *M. indica* extracts showed some dermal and eye irritation potential in the rabbit, these effects were found to be reversible. Earlier studies demonstrated that MSBE was nonirritating to the skin or in the eye (Garrido et al., 2009). Further, MSBE showed no or minimal irritation following rectal or vaginal application, respectively (Garrido et al., 2009). Prado et al. (2015) conducted a battery of acute and 28‐day toxicity tests on 92% pure mangiferin, finding no acute dermal toxicity in mice and rats. The authors reported some treatment‐related effects in the high‐dose 1,000 mg/kg group, whereas 250 and 500 mg/kg groups showed no adverse effects. Specifically at 1000 mg/kg, histopathological alterations such as vacuolar degeneration, necrosis, and increment of apoptosis of the acinar cells were observed in the exocrine pancreas of rats. In contrast, the 28‐d toxicity test with LI12542F6 showed no pancreatic effects of 500, 1000, or 1500 mg/kg BW in female rats but 1 incidence of acinar degeneration/atrophy in a control female rat. While 1 notable finding of acinar degeneration/atrophy at the 1500 mg/kg BW in a single male rat was observed, no similar findings were seen in the pancreas of male or female rats at any dose in the 90‐day subchronic study. As LI12542F6 is an herbal blend containing extracts from two plants, it has a much lower concentration of mangiferin than the 92% pure mangiferin used in the Prado study. This herbal combination showed a NOAEL value greater than 2,000 mg/kg body weight in the 90‐day study described above.

In addition to the toxicological studies described in this paper, a randomized, double‐blind, placebo‐controlled study has been conducted with 40 male subjects who consumed either LI12542F6 (two capsules of 325 mg each) or placebo (two capsules) every morning for 56 days. No adverse events were reported, and there were no adverse outcomes in vital signs and standard hematology, biochemical, clinical chemistry, and urinalysis laboratory values (Gora, Manikyeswararao, Alluri, & Davis, 2019). In addition, there is a significant history of human consumption of both plants *S. indicus* and *M. indica* (Batool et al., 2018; Coe & Anderson, 1996; George et al., 2017; Guevara et al., 2004; Masud Parvez, 2016; Prado et al., 2015; Ramachandran, 2013; Sharma et al., 2001; Yoshimi et al., 2001) which are the principal ingredients in LI12542F6.

## 5 CONCLUSION

LI12542F6 is shown to have no genotoxic potential and only limited irritation properties in a comprehensive series of genotoxicity and animal toxicity studies. The NOAEL from the 90‐day rat study is >2,000 mg/kg bw per day.

## CONFLICT OF INTEREST

ERN is an independent toxicologist. VKA and SD are employees of Laila Nutraceuticals, India. BAD is an employee of PLT Health Solutions, NJ, USA.

## ETHICAL STATEMENT

The study's protocols and procedures were ethically reviewed and approved by the Institutional Animal Ethics Committee (IAEC), IAEC approval No. LN/IAEC/TOX/LN170803, in compliance with OECD Guideline No. 408, and the Committee for the Purpose of Control and Supervision of Experiments on Animals (CPCSEA), Government of India (CPCSEA Registration No of the Test Facility: 1668/PO/RcBi/S/12/CPCSEA).
