# Supplementary material for: Toxicological studies on the botanical supplement LI12542F6 containing extracts of Sphaeranthus indicus flower heads and Mangifera indica (mango tree) bark
Source: Food Sci Nutr. 2019 Jan 29;7(2):817–33. doi: 10.1002/fsn3.931 (PMC6392882; doi:10.1002/fsn3.931)
Supplement: Supplementary file 1 [file FSN3-7-817-s001.docx]

# Supplementary Data

**Toxicological studies on the botanical supplement LI12542F6 containing extracts of *Sphaeranthus indicus***

**flower heads and *Mangifera indica* (mango tree) bark.**

Earle R. Nestmann1, Venkata Krishnaraju Alluri2, Sundararaju Dodda2 and Barbara A. Davis3* 1Health Science Consultants, Mississauga, Canada

2 Laila Nutraceuticals R&D Center, Vijayawada-520007, India

3PLT Health Solutions Inc., Morristown, NJ 07960

*Corresponding Author: Barbara A. Davis, PhD, RD [barbara@plthealth.com](mailto:barbara@plthealth.com)

119 Headquarters Plaza, Morristown, NJ 06019

Phone: 973-984-0900 ext. 252

Table 1: Preliminary observation on genotoxicity potential of LI12542F6 in reverse mutation assay in presence or in absence of metabolic activation

| Treatment (µg/plate) | TA100 Revertant colonies/plate | | | | | | | | | |
| --- | --- | --- | --- | --- | --- | --- | --- | --- | --- | --- |
|  | Presence of S9 | | | | | Absence of S9 | | | | |
|  | R1 | R2 | Mean | Backgro-und lawn | Precipi-tation | R1 | R2 | Mean | Backgro-und lawn | Precipi-tation |
| Vehicle control  DMSO | 86 | 84 | 85 | 4+ | Nil | 89 | 90 | 90 | 4+ | Nil |
| 50 | 85 | 83 | 84 | 4+ | Nil | 85 | 86 | 86 | 4+ | Nil |
| 100 | 77 | 82 | 80 | 4+ | Nil | 83 | 80 | 82 | 4+ | Nil |
| 200 | 70 | 72 | 71 | 4+ | Nil | 68 | 70 | 69 | 4+ | Nil |
| 400 | 71 | 68 | 70 | 4+ | Nil | 69 | 71 | 70 | 4+ | Nil |
| 800 | 66 | 70 | 68 | 4+ | Nil | 69 | 65 | 67 | 4+ | Nil |
| 1600 | 67 | 69 | 68 | 4+ | Nil | 67 | 64 | 66 | 4+ | Nil |
| 3200 | 64 | 66 | 65 | 4+ | Nil | 62 | 65 | 64 | 4+ | Nil |
| 5000 | 45 | 47 | 46 | 3+ | Nil | 48 | 50 | 49 | 3+ | Nil |

R1, R2- replicates; 4+, non-toxic; 3+, slightly toxic

Table 2: Observation on confirmatory mutation assay evaluating genotoxicity potential of LI12542F6 in presence of metabolic activation

| Treatment  (µg/plate) | No. of revertants/plate | | | | | | | | | |
| --- | --- | --- | --- | --- | --- | --- | --- | --- | --- | --- |
|  | TA98 | | TA100 | | TA1535 | | TA1537 | | WP2*uvrA*(pkm101) | |
|  | Mean ± SD | Ratio^b^ | Mean ± SD | Ratio^b^ | Mean ± SD | Ratio^b^ | Mean ± SD | Ratio^b^ | Mean ± SD | Ratio^b^ |
| Vehicle control  DMSO | 28 ± 3 | NA | 90 ± 3 | NA | 14 ± 2 | NA | 12 ± 2 | NA | 132 ± 5 | NA |
| 100 | 25 ± 3 | 0.89 | 80 ± 5 | 0.89 | 10 ± 3 | 0.71 | 9 ± 3 | 0.75 | 128 ± 3 | 0.97 |
| 266 | 22 ± 2 | 0.79 | 72 ± 3 | 0.80 | 11 ± 2 | 0.79 | 8 ± 2 | 0.67 | 127 ± 3 | 0.96 |
| 707 | 21 ± 2 | 0.75 | 69 ± 4 | 0.77 | 8 ± 1 | 0.57 | 8 ± 2 | 0.67 | 126 ± 3 | 0.95 |
| 1880 | 18 ± 2 | 0.64 | 68 ± 3 | 0.76 | 8 ± 3 | 0.57 | 6 ± 1 | 0.50 | 125 ± 4 | 0.95 |
| 5000 | 16 ± 2 | 0.57 | 47 ± 3 | 0.52 | 5 ± 1 | 0.36 | 5 ± 2 | 0.42 | 112 ± 3 | 0.85 |
| Positive control | 571^c^ ± 8 | 20.39 ^c^ | 882^c^ ± 12 | 9.80 ^c^ | 151 ^c^ ± 12 | 10.79 ^c^ | 153^c^ ± 9 | 12.75 ^c^ | 590 ^d^ ± 6 | 4.47 ^d^ |

^b^ Ratio of treated / vehicle control (mean revertants per plate)

^c^ TA98, TA100, TA1535, TA1537 : 2 - Aminoanthracene (4 µg/plate)

^d^ WP2*uvrA*(pkm101): 2-Aminoanthracene (30 µg/plate)

NA: Not applicable; SD: Standard deviation

Table 3: Observation on confirmatory mutation assay evaluating genotoxicity potential of LI12542F6 in absence of metabolic activation

| Treatment  (µg/plate) | No. of revertants/plate | | | | | | | | | |
| --- | --- | --- | --- | --- | --- | --- | --- | --- | --- | --- |
|  | TA98 | | TA100 | | TA1535 | | TA1537 | | WP2*uvrA*(pkm101) | |
|  | Mean ± SD | Ratio^b^ | Mean ± SD | Ratio^b^ | Mean ± SD | Ratio^b^ | Mean ± SD | Ratio^b^ | Mean ± SD | Ratio^b^ |
| Vehicle control  DMSO | 27 ± 2 | NA | 91 ± 3 | NA | 13 ± 3 | NA | 12 ± 3 | NA | 135 ± 2 | NA |
| 100 | 23 ± 4 | 0.85 | 81 ± 2 | 0.89 | 12 ± 2 | 0.92 | 9 ± 1 | 0.75 | 127 ± 2 | 0.94 |
| 266 | 21 ± 3 | 0.78 | 76 ± 3 | 0.84 | 9 ± 2 | 0.69 | 8 ± 2 | 0.67 | 128 ± 3 | 0.95 |
| 707 | 21 ± 2 | 0.78 | 70 ± 3 | 0.77 | 8 ± 2 | 0.62 | 8 ± 2 | 0.67 | 124 ± 2 | 0.92 |
| 1880 | 18 ± 4 | 0.67 | 67 ± 3 | 0.74 | 8 ± 3 | 0.62 | 6 ± 1 | 0.50 | 125 ± 4 | 0.93 |
| 5000 | 16 ± 3 | 0.59 | 46 ± 2 | 0.51 | 5 ± 1 | 0.38 | 5 ± 1 | 0.42 | 113 ± 5 | 0.84 |
| Positive control | 277^c^ ± 14 | 10.26 ^c^ | 765^d^ ± 169 | 8.41 ^d^ | 151 ^d^ ± 6 | 11.62 ^d^ | 151^e^ ± 7 | 12.58 ^e^ | 563 ^f^ ± 8 | 4.17 ^f^ |

^b^ Ratio of treated / vehicle control (mean revertants per plate) ;

^c^ TA98 : 2-Nitrofluorene (2 µg/plate) ;

^d^ TA100,TA 1535:Sodium azide(1 µg/plate);

^e^ TA1537:9-Aminoaoacridine(50 µg/plate) ;

^f^ WP2*uvrA*(pkm101): 4-Nitroquinoline -N -Oxide (4µg/plate)

NA: Not applicable; SD: Standard deviation

| **Parameter** | **Group & Dose (mg/kg b.wt/day)** | | | |
| --- | --- | --- | --- | --- |
|  | **G1 - 0** | **G2 - 1000** | **G3 - 1500** | **G4 - 2000** |
|  | **Mean ± SD** | **Mean ± SD** | **Mean ± SD** | **Mean ± SD** |
| RBC (106  cells/μL) | 9.01 ± 0.57 | 8.94 ± 0.33 | 8.43 ± 0.96 | 8.66 ± 0.39 |
| Hemoglobin (g/dL) | 15.63 ± 0.62 | 15.47 ± 0.53 | 15.18 ± 1.01 | 15.54 ± 0.86 |
| Hematocrit (%) | 47.83 ± 1.79 | 47.93 ± 1.26 | 46.66 ± 3.43 | 48.02 ± 2.49 |
| MCV (fL) | 53.17 ± 2.3 | 53.68 ± 1.49 | 55.65 ± 3.01 | 55.51 ± 2.33 |
| MCH (pg) | 17.40 ± 0.77 | 17.32 ± 0.28 | 18.12 ± 1.28 | 17.99 ± 0.83 |
| MCHC (g/dL) | 32.73 ± 0.57 | 32.29 ± 0.69 | 32.55 ± 1.59 | 32.40 ± 0.47 |
| Platelets (103 cells/μL) | 893.10 ± 162.3 | 980.80 ± 81.51 | 1053.00 ± 160.10*↑ | 948.40 ± 136.00 |
| WBC (103  cells/μL) | 8.18 ± 1.8 | 7.33 ± 1.22 | 6.96 ± 1.24 | 7.01 ± 1.79 |
| Neutrophils (%) | 16.37 ± 6.22 | 17.66 ± 3.10 | 19.24 ± 6.39 | 14.35 ± 3.62 |
| Lymphocytes (%) | 78.73 ± 6.73 | 78.04 ± 2.93 | 76.77 ± 5.86 | 81.03 ± 3.47 |
| Monocytes (%) | 1.94 ± 0.59 | 1.84 ± 0.56 | 1.65 ± 0.37 | 1.92 ± 0.53 |
| Eosinophils (%) | 1.31 ± 0.53 | 0.92 ± 0.33 | 1.01 ± 0.29 | 1.10 ± 0.69 |
| Basophils (%) | 0.20 ± 0.08 | 0.15 ± 0.07 | 0.20 ± 0.08 | 0.17 ± 0.08 |
| LUC (%) | 1.43 ± 0.35 | 1.34 ± 0.25 | 1.12 ± 0.30 | 1.42 ± 0.36 |
| Retic (%) | 0.50 ± 0.48 | 0.74 ± 0.43 | 0.90 ± 0.55 | 0.47 ± 0.42 |
| CT (Sec) | 135.90 ± 21.47 | 150.80 ± 23.93 | 147.70 ± 25.22 | 134.60 ± 18.39 |

n = 10 in control, 1000 mg/kg, 1500 mg/kg, and 2000 mg/kg LI12542F6 groups. *↑;Significantly higher than the

control group, p<0.05.

| **Parameter** | **Group & Dose (mg/kg b.wt/day)** | | | |
| --- | --- | --- | --- | --- |
|  | **G1 - 0** | **G2 - 1000** | **G3 - 1500** | **G4 - 2000** |
|  | **Mean ± SD** | **Mean ± SD** | **Mean ± SD** | **Mean ± SD** |
| RBC (106  cells/μL) | 8.33 ± 0.32 | 7.84 ± 0.37 | 7.81 ± 0.69 | 7.78 ± 0.70 |
| Hemoglobin (g/dL) | 15.32 ± 0.28 | 14.31 ± 0.62*↓ | 14.74 ± 1.05 | 14.73 ± 1.01 |
| Hematocrit (%) | 46.96 ± 1.32 | 44.16 ± 1.54*↓ | 45.48 ± 3.49 | 44.98 ± 2.61 |
| MCV (fL) | 56.44 ± 2.92 | 56.34 ± 1.26 | 58.39 ± 2.63 | 58.06 ± 3.57 |
| MCH (pg) | 18.40 ± 0.67 | 18.25 ± 0.42 | 18.94 ± 0.78 | 18.99 ± 1.05 |
| MCHC (g/dL) | 32.62 ± 0.60 | 32.4 ± 0.42 | 32.42 ± 0.39 | 32.75 ± 0.59 |
| Platelets (103 cells/μL) | 955.20 ± 113.80 | 1135.00 ± 141.29*↑ | 1062.70 ± 174.24 | 1049.50 ± 140.66 |
| WBC (103  cells/μL) | 5.06 ± 1.07 | 5.39 ± 1.44 | 5.11 ± 1.40 | 4.79 ± 1.58 |
| Neutrophils (%) | 12.72 ± 4.26 | 12.57 ± 4.14 | 15.76 ± 6.90 | 11.79 ± 2.67 |
| Lymphocytes (%) | 82.19 ± 4.97 | 82.92 ± 4.72 | 78.90 ± 7.23 | 83.43 ± 3.30 |
| Monocytes (%) | 1.93 ± 0.65 | 1.80 ± 0.56 | 2.39 ± 0.58 | 1.96 ± 0.56 |
| Eosinophils (%) | 1.63 ± 0.62 | 1.29 ± 0.68 | 1.57 ± 0.59 | 1.25 ± 0.38 |
| Basophils (%) | 0.18 ± 0.06 | 0.16 ± 0.05 | 0.19 ± 0.08 | 0.20 ± 0.07 |
| LUC (%) | 1.37 ± 0.38 | 1.24 ± 0.20 | 1.25 ± 0.39 | 1.40 ± 0.50 |
| Retic (%) | 0.73 ± 0.38 | 0.70 ± 0.49 | 0.83 ± 0.52 | 1.00 ± 0.40 |
| CT (Sec) | 126.10 ± 13.21 | 137.80 ± 17.01 | 135.70 ± 19.25 | 127.40 ± 17.94 |

n = 10 in control, 1000 mg/kg, 1500 mg/kg, and 2000 mg/kg LI12542F6 groups.

*↓: Significantly lower than the control group, p<0.05, *↑: Significantly higher than the control group, p<0.05

| **Parameter** | **Group & Dose (mg/kg b.wt/day)** | | | |
| --- | --- | --- | --- | --- |
|  | **G1 - 0** | **G2 - 1000** | **G3 - 1500** | **G4 - 2000** |
|  | **Mean ± SD** | **Mean ± SD** | **Mean ± SD** | **Mean ± SD** |
| Glu (mg/dL) | 111.70 ± 9.72 | 107.60 ± 13.25 | 108.30 ± 14.87 | 120.10 ± 13.83 |
| BUN (mg/dL) | 20.90 ± 3.11 | 21.50 ± 4.38 | 22.50 ± 2.32 | 22.10 ± 3.51 |
| Ur (mg/dL) | 44.60 ± 6.26 | 46.20 ± 9.22 | 48.00 ± 5.01 | 47.20 ± 7.51 |
| Crea (mg/dL) | 0.59 ± 0.05 | 0.55 ± 0.04 | 0.58 ± 0.06 | 0.58 ± 0.04 |
| T.Chol (mg/dL) | 66.70 ± 10.39 | 61.80 ± 9.76 | 63.20 ± 8.02 | 59.90 ± 5.15 |
| Trig (mg/dL) | 79.70 ± 18.17 | 84.50 ± 29.22 | 82.50 ± 34.21 | 71.80 ± 19.97 |
| T.Bil (mg/dL) | 0.11 ± 0.04 | 0.08 ± 0.02*↓ | 0.07 ± 0.03**↓ | 0.09 ± 0.02 |
| AST (U/L) | 102.35 ± 18.95 | 87.40 ± 9.94 | 90.70 ± 9.96 | 80.20 ± 17.34**↓ |
| ALT (U/L) | 56.30 ± 13.37 | 48.00 ± 7.67 | 49.00 ± 8.50 | 45.00 ± 12.17 |
| ALP (U/L) | 87.70 ± 17.1 | 97.40 ± 29.78 | 72.80 ± 16.14 | 69.80 ± 9.99 |
| TP (g/dL) | 7.01 ± 0.34 | 6.82 ± 0.28 | 6.75 ± 0.30 | 6.72 ± 0.32 |
| Alb (g/dL) | 3.82 ± 0.1 | 3.77 ± 0.13 | 3.78 ± 0.16 | 3.79 ± 0.12 |
| Ca (mg/dL) | 10.81 ± 0.24 | 10.71 ± 0.32 | 10.54 ± 0.41 | 10.56 ± 0.42 |
| Phos (mg/dL) | 5.16 ± 0.37 | 5.08 ± 0.69 | 5.19 ± 0.84 | 5.04 ± 0.45 |
| Na (mmoL/L) | 137.86 ± 1.55 | 140.06 ± 0.87*↑ | 139.69 ± 1.86*↑ | 139.12 ± 2.10 |
| K (mmoL/L) | 3.89 ± 0.24 | 3.83 ± 0.28 | 3.80 ± 0.25 | 4.01 ± 0.22 |
| Cl (mmoL/L) | 106.48 ± 1.36 | 107.34 ± 1.33 | 108.01 ± 2.07 | 107.19 ± 1.75 |

n = 10 in control, 1000 mg/kg, 1500 mg/kg, and 2000 mg/kg LI12542F6 groups. *↓: Significantly lower than the control group; *↑: Significantly higher than the control group, p<0.05; **↓: Significantly lower than the control group, p<0.01.

| **Parameter** | **Group & Dose (mg/kg b.wt/day)** | | | |
| --- | --- | --- | --- | --- |
|  | **G1 - 0** | **G2 - 1000** | **G3 - 1500** | **G4 - 2000** |
|  | **Mean ± SD** | **Mean ± SD** | **Mean ± SD** | **Mean ± SD** |
| Glu (mg/dL) | 91.10 ± 15.88 | 97.50 ± 11.95 | 92.40 ± 13.70 | 99.60 ± 19.33 |
| BUN (mg/dL) | 22.40 ± 3.34 | 24.10 ± 3.45 | 24.80 ± 4.64 | 23.80 ± 3.16 |
| Ur (mg/dL) | 47.80 ± 7.08 | 51.70 ± 6.96 | 52.50 ± 9.68 | 50.60 ± 6.74 |
| Crea (mg/dL) | 0.68 ± 0.07 | 0.68 ± 0.06 | 0.69 ± 0.07 | 0.66 ± 0.06 |
| T.Chol (mg/dL) | 45.60 ± 17.01 | 54.50 ± 9.86 | 59.10 ± 12.74 | 49.20 ± 8.63 |
| Trig (mg/dL) | 72.80 ± 33.63 | 67.10 ± 23.14 | 79.20 ± 28.28 | 68.0 ± 26.82 |
| T.Bil (mg/dL) | 0.12 ± 0.08 | 0.10 ± 0.07 | 0.08 ± 0.04 | 0.07 ± 0.03 |
| AST (U/L) | 104.60 ± 18.11 | 83.30 ± 10.29*↓ | 83.90 ± 17.32*↓ | 84.70 ± 13.26*↓ |
| ALT (U/L) | 50.20 ± 13.36 | 41.20 ± 9.66 | 40.50 ± 15.62 | 36.40 ± 8.57*↓ |
| ALP (U/L) | 42.80 ± 12.93 | 35.20 ± 12.49 | 32.20 ± 10.51 | 33.10 ± 12.63 |
| TP (g/dL) | 6.89 ± 0.32 | 6.96 ± 0.42 | 7.11 ± 0.57 | 6.78 ± 0.64 |
| Alb (g/dL) | 4.02 ± 0.20 | 4.09 ± 0.26 | 4.21 ± 0.30 | 4.03 ± 0.37 |
| Ca (mg/dL) | 10.45 ± 0.35 | 10.68 ± 0.47 | 10.72 ± 0.36 | 10.47 ± 0.70 |
| Phos (mg/dL) | 4.21 ± 0.47 | 4.00 ± 0.63 | 4.11 ± 0.38 | 4.21 ± 0.68 |
| Na (mmoL/L) | 138.12 ± 0.87 | 138.63 ± 0.87 | 140.77 ± 2.53*↑ | 140.24 ± 2.93 |
| K (mmoL/L) | 3.52 ± 0.19 | 3.73 ± 0.21 | 3.49 ± 0.22 | 3.36 ± 0.19 |
| Cl (mmoL/L) | 107.92 ± 1.07 | 107.62 ± 1.06 | 109.29 ± 2.35 | 108.59 ± 2.86 |

n = 10 in control, 1000 mg/kg, 1500 mg/kg, and 2000 mg/kg LI12542F6 groups. *↓: Significantly lower than the control group, p<0.05. *↑: Significantly higher than the control group, p<0.05.

| **Organs / Tissue** | **Group & Dose (mg/kg b.wt/day)** | | | |
| --- | --- | --- | --- | --- |
|  | **G1 - 0** | **G2 - 1000** | **G3 - 1500** | **G4 - 2000** |
|  | **Mean ± SD** | **Mean ± SD** | **Mean ± SD** | **Mean ± SD** |
| Liver | 10.4979 ± 2.6394 | 11.0540 ± 1.7602 | 10.7616 ± 1.3883 | 11.1500 ± 1.3900 |
| Kidneys | 2.4582 ± 0.5732 | 2.6560 ± 0.3556 | 2.399 ± 0.3287 | 2.6648 ± 0.3920 |
| Adrenal Glands | 0.0520 ± 0.0114 | 0.0539 ± 0.0077 | 0.0513 ± 0.0102 | 0.0540 ± 0.0076 |
| Heart | 1.0997 ± 0.2077 | 1.0917 ± 0.1619 | 0.9823 ± 0.1081 | 1.0147 ± 0.1003 |
| Brain | 1.9117 ± 0.1964 | 1.9737 ± 0.1150 | 1.9720 ± 0.1475 | 2.0236 ± 0.1105 |
| Spleen | 0.6938 ± 0.1236 | 0.7009 ± 0.0796 | 0.7041 ± 0.0877 | 0.7035 ± 0.098 |
| Thymus | 0.3939 ± 0.1174 | 0.4507 ± 0.1086 | 0.4222 ± 0.0994 | 0.3692 ± 0.0956 |
| Testes | 3.5503 ± 0.4078 | 3.5058 ± 0.3626 | 3.3823 ± 0.5263 | 3.622 ± 0.5304 |
| Epididymides | 1.3202 ± 0.2573 | 1.2722 ± 0.1155 | 1.1938 ± 0.1049 | 1.3003 ± 0.1757 |
| SV-CG and Prostate Gland | 2.9458 ± 0.5755 | 2.6972 ± 0.1996 | 2.5907 ± 0.2495 | 2.8475 ± 0.5162 |

n = 10 in control, 1000 mg/kg, 1500 mg/kg, and 2000 mg/kg LI12542F6 groups.

| **Organs / Tissue** | **Group & Dose (mg/kg b.wt/day)** | | | |
| --- | --- | --- | --- | --- |
|  | **G1 - 0** | **G2 - 1000** | **G3 - 1500** | **G4 - 2000** |
|  | **Mean ± SD** | **Mean ± SD** | **Mean ± SD** | **Mean ± SD** |
| Liver | 6.1438 ± 1.3877 | 6.7454 ± 0.9239 | 7.2258 ± 0.8220 | 6.7827 ± 1.0276 |
| Kidneys | 1.489 ± 0.1624 | 1.5909 ± 0.1856 | 1.6421 ± 0.2713 | 1.6062 ± 0.2857 |
| Adrenal Glands | 0.0667 ± 0.0129 | 0.0703 ± 0.0077 | 0.0691 ± 0.009 | 0.0718 ± 0.0108 |
| Heart | 0.6604 ± 0.0767 | 0.7132 ± 0.0624 | 0.6985 ± 0.0697 | 0.7055 ± 0.0929 |
| Brain | 1.8267 ± 0.0932 | 1.8472 ± 0.0784 | 1.8733 ± 0.1533 | 1.8571 ± 0.125 |
| Spleen | 0.4908 ± 0.0578 | 0.5485 ± 0.0553 | 0.5663 ± 0.1156 | 0.5823 ± 0.1233 |
| Thymus | 0.3323 ± 0.111 | 0.396 ± 0.0758 | 0.4056 ± 0.1021 | 0.3928 ± 0.1195 |
| Uterus with Cervix | 0.8018 ± 0.3615 | 0.8021 ± 0.3627 | 0.591 ± 0.1066 | 0.5927 ± 0.0898 |
| Ovaries | 0.1356 ± 0.0313 | 0.1598 ± 0.0292 | 0.1428 ± 0.0345 | 0.154 ± 0.0395 |

n = 10 in control, 1000 mg/kg, 1500 mg/kg, and 2000 mg/kg LI12542F6 groups.
